# Early risk predictors of acute kidney injury and short-term survival during Impella support in cardiogenic shock

**DOI:** 10.1038/s41598-024-68376-w

**Published:** 2024-07-30

**Authors:** Nikolaos Patsalis, Julian Kreutz, Giorgos Chatzis, Styliani Syntila, Maryana Choukeir, Bernhard Schieffer, Birgit Markus

**Affiliations:** Department of Cardiology, Angiology, and Intensive Care Medicine, University Hospital, Philipps University of Marburg, Baldinger Str., 35043 Marburg, Germany

**Keywords:** Cardiogenic shock, Acute kidney injury (AKI), Left ventricular Impella, Predictors of AKI, Physiology, Nephrology, Predictive markers, Cardiology, Cardiac device therapy, Risk factors, Cardiovascular diseases

## Abstract

Acute kidney injury (AKI) is one of the most frequent and prognostic-relevant complications of cardiogenic shock (CS) complicating myocardial infarction (MI). Mechanical circulatory assist devices (MCS) like left ventricular Impella microaxial pump have increasingly been used in the last decade for stabilization of hemodynamics in those patients. Moreover, a protective effect of Impella on renal organ perfusion could recently be demonstrated. However, data identifying early risk predictors for developing AKI during Impella support in CS are rare. Data of hemodynamics and renal function from 50 Impella patients (January 2020 and February 2022) with MI-related CS (SCAI stage C), were retrospectively analyzed using e.g. multivariate logistic regression analysis as well as Kaplan–Meier curves and Cox regression analysis. 30 patients (60%) developed AKI. Central venous pressure as an indicator for venous congestion (OR 1.216, p = 0.02), GFR at admission indicating existing renal damage (OR 0.928, p = 0.002), and reduced central venous oxygen saturation (SvO_2_) as a marker for decreased tissue perfusion (OR 0.930, p = 0.029) were independently associated with developing an AKI. The 30-day mortality rate was significantly higher in patients with AKI stage 3 (Stage 1: 0%, Stage 2: 0%, Stage 3; 41.6%, p = 0.014) while AKI stage 3 (HR 0.095, p = 0.026) and norepinephrine dosage (HR 1.027, p = 0.008) were independent predictors for 30-day mortality. AKI as a complication of MI-related CS occurs frequently with a major impact on prognosis. Venous congestion, reduced tissue perfusion, and an already impaired renal function are independent predictors of AKI. Thus, timely diagnostics and a focused treatment of the identified factors could improve prognosis and outcome.

## Introduction

Cardiogenic shock (CS) is a feared complication in approximately 10% of acute myocardial infarction (MI) with a 30-day mortality of up to 40% ^1^. The impairment of myocardial contractility in CS is the most relevant pathophysiological problem. A reduced cardiac output (CO) results in reduced blood pressure, which in turn further impairs organ and coronary perfusion and, consequently, myocardial contractility. As part of the development of this so-called downward shock spiral, tissue and end-organ perfusion thus deteriorates considerably with corresponding consequences in terms of organ failure ^2^.

Acute kidney injury (AKI) is one of the most frequent complications of a reduced CO in CS with a cumulative risk of 20–35% and a significant impact on prognosis ^3^. Patients with AKI are accompanied by a 12 times greater 90-day mortality and the additional necessity of renal replacement therapy (RRT) further increases intrahospital mortality (62% vs. 46%) ^3–6^. To stabilize hemodynamics, high dosages of vasopressors are very often being substituted. However, vasoconstrictors only achieve a pseudo normalization of blood pressure while the systemic vascular resistance and hence the cardiac afterload rise, which in turn further burdens myocardial contractility and organ perfusion. During the past years, percutaneous left ventricular assist devices (pLVAD) such as the Impella micro axial pump have increasingly been used to improve CO, reduce vasopressor dosages, and thus optimize tissue and end-organ perfusion and at least function ^7–9^. While some studies have investigated the prevalence of AKI in CS, data analyzing patients with CS being supported with Impella are rare. The goal of this study was to evaluate early risk factors and define subsequent predictor characteristics for developing AKI during Impella support in CS as well as to identify potential therapeutic options during Impella support concerning the predisposing factors of AKI.

## Methods

### Study design

This was a retrospective data analysis of patients with CS and Impella support, admitted to the University Hospital of Marburg from January 2020 to February 2022. To be included in this analysis, patients had to present with CS due to myocardial infarction, corresponding to stage C of the SCAI classification. Data of patients with single kidney and underlying autoimmune or polycystic kidney disease were not included in this analysis.

Risk predictors for developing AKI and the influence of AKI on survival were retrospectively evaluated.

The definition of CS predisposed the presence of the following three factors: (1) impaired systolic blood pressure < 90 mmHg, longer than 30 min or necessary catecholamine support to maintain systolic blood pressure ≥ 90 mmHg, (2) onset of pulmonary congestion (3) clinical evidence of burdened end-organ perfusion (one or more of the following: serum lactate above 2.0 mmol/L, cold and wet cutis, urine output below 30 ml/h, pathological mental function), corresponding to SCAI stage C ^10^.

AKI was defined as an elevation of serum creatinine ≥ 0.3 mg/dl during the initial 48 h after admission to the hospital according to the *Kidney Disease Improving Global Outcomes* (KDIGO) criteria ^11^. Urine output criteria were not considered since the initial administration of diuretics could represent a relevant confounder ^12^. For patients without any recent data regarding creatinine levels (n = 3), we assessed baseline creatinine by implementing an estimated glomerular filtration rate (eGFR) of 75 ml/min/1.73 m^2^ (eGFR approach) ^13–15^. Stages of chronic kidney disease (CKD) were determined by assessment of baseline (admission to the hospital) creatinine levels according to current KDIGO guidelines ^16^.

In all patients, the implantation of Impella and the pulmonary artery catheter (PAC) for invasive hemodynamic measurements were performed within the first 120 min after admission according to standard operating procedures in the catheterization laboratory before the PCI procedure of the culprit lesion took place (suppl. Table 1). Before implantation of the Impella, the LVEF was measured using echocardiography.

### Invasive hemodynamic measurement

PAC was implemented to obtain hemodynamic parameters like cardiac output (CO), systolic arterial pulmonary pressure (sPAP), diastolic arterial pulmonary pressure (dPAP) and mean arterial pulmonary pressure (meanPAP), pulmonary capillary wedge pressure (PCWP), central venous pressure (CVP), central venous oxygen saturation (SvO_2_), systemic vascular resistance (SVR) and pulmonary artery pulsatility index (PAPi). Parameters were taken within the first 24 h after admission to the hospital, after the patient´s arrival on the ICU with ongoing Impella support.

### Comorbidities, clinical parameters, and ICU scores

Comorbidities and clinical and treatment-related parameters, like systolic, diastolic, and mean arterial pressure, heart rate, catecholamine dosages and fluids, laboratory parameters including serum creatinine and GFR and lactate were registered and evaluated during the in-hospital stay. The common ICU scores (SAPS II, SOFA) and the Horowitz Index were documented within the first 24 h after admission.

### Clinical outcomes

Primary endpoint was the development of AKI. Risk factors for the development of AKI, all-cause mortality and risk factors for mortality were defined as secondary endpoints.

### Statistical analysis

This was a retrospective data analysis. Data are presented as absolute variables and percentages (%) for categorical variables and either median with interquartile range (IQR: 25th–75th percentile) or mean with standard deviation according to the distribution of the variables. Normality was assessed by using Kolmogorov–Smirnov as well as the Shapiro–Wilk test. After testing for normal distribution, the Student’s t-test or Mann–Whitney test was implemented to test for differences between various characteristics. For categorical variables, Fisher’s exact test or chi-square test was used, as appropriate. We implemented binary logistic regression analysis to identify variables associated with AKI. Considering significant associations of univariate analysis (p < 0.1 accepted for retention), multivariate logistic regression with backward elimination and probability of occurrence of 0.05 and removal of 0.10 was performed to identify independent predictors. Pearson's correlation was implemented to analyze for correlations with a p < 0.05 considered statistically significant. Kaplan–Meier curves with log-rank (Mantel-Cox) test was used to analyze 30-day survival. Cox regression analysis was performed to identify independent predictors considering variables with significant association at univariate analysis (retention p < 0.1). All analyses were made using SPSS 28 (IBM Corp., USA) and GraphPad Prism 8.0. A two-sided p-value < 0.05 was considered statistically significant.

### Ethics approval and consent to participate

The data analysis was performed according to the Declaration of Helsinki and was approved by the Ethics Committee of the University Hospital of Marburg (protocol code: study 136/17, date of approval: 11 October 2019). Informed consent was acquired from all patients involved.

## Results

### Overall cohort

Data from 50 patients were analyzed. The demographics, baseline characteristics, and relevant comorbidities of the overall cohort are displayed in Table 1. All patients presented with shock stadium C according to the SCAI classification. Only Impella MCS was used in this study cohort. None of the patients' hemodynamic situation deteriorated during Impella support and none suffered cardiac arrest. The study population had a mean age of 67 ± 13 years with 74% male individuals. 18 (36%) of the patients were accompanied by a 3-vessel coronary heart disease (CHD), 19 (38%) had a 2-vessel CHD and in 13 (26%) a 1-vessel CHD was diagnosed. Culprit lesions were in 4 patients (8%) in the left main coronary artery (LMCA), in 19 patients (38%) in the left anterior descending (LAD), in 6 patients (12%) in the LAD and first diagonal branch, in 4 patients (8%) in the LAD and in the second diagonal branch, in 6 patients (12%) in the intermediary branch, in 8 patients (16%) in the LCX, and in 3 patients (6%) in the right posterolateral branch. None of the patients presented a right ventricular infarction (Suppl. Table 1). Every patient underwent coronary angiography with appropriate percutaneous coronary intervention and implantation of left ventricular Impella (CP) under fluoroscopic control in the catheter laboratory. In all patients, arterial access was established via the femoral artery. 43 (86%) patients had a medical history of arterial hypertension (aHT), 35 (70%) were accompanied by known dyslipidemia, and in 19 (38%) patients diabetes mellitus was diagnosed. In the overall cohort, the mean LVEF was 37 ± 12% and the mean duration of Impella support was 9 ± 6 days, without any significant differences in the subgroups (AKI, Non-AKI). No relevant hemolysis (significant hemoglobine decrease, macrohematuria, bilirubin increase, free hemoglobine) could be detected. Data on renal function and hemodynamic data of the overall cohort are shown in Tables 2 and 3 respectively.

**Table 1:** Medical history and baseline characteristics of the overall cohort, in non-AKI and AKI patients.

	Total	Non-AKI (n = 20)	AKI (n = 30)	p value
Demographics, characteristics and comorbidities				
Age	64 ± 13	64.5 ± 14.7	68.9 ± 11.7	0.125
Female n (%)	13 (26)	4 (31)	9 (19)	0.43
Male n (%)	37 (74)	16 (69)	21 (81)	0.43
BMI	26.4 ± 3.4	24.34 ± 6.7	26.94 ± 3.3	0.06
History of aHT n (%)	43 (86.0)	16 (80.0)	27 (90.0)	0.318
History of dyslipidemia n (%)	35 (70.0)	12 (63.2)	23 (76.7)	0.309
History of DM n (%)	19 (38.0)	6 (31.5)	13 (43.9)	0.411
CHD n (%)	50 (100%)			–
1-vessel CHD	13 (26)	6 (31.6)	12 (40)	
2-vessel CHD	19 (38)	7 (36.8)	6 (20)	
3-vessel-CHD	18 (36)	6 (31.6)	12 (40)	
LVEF (%)	37 ± 12	42 ± 11.2	37.5 ± 11.6	0.092
COPD n (%)	9	7(75.9)	2 (9.5)	0.184
PAH n (%)	0	–	–	–
SOFA score (pts. (IQR))	10 (5)	6 (6)	11 (5)	0.001
SAPS II (pts.)	44.36 ± 15.03	36.3 ± 11.9	50.17 ± 14.51	0.001
Horowitz index (mmHg)	240.78 ± 94.67	274 ± 98.9	218.6 ± 86.3	0.02
Impella flow min/max (l/min)	1.65 ± 0.65/2.48 ± 0.63	1.48 ± 0.39/2.01 ± 0.34	1.75 ± 0.76/2.2 ± 0.5	0.07

*aHT* arterial hypertension, *AKI* acute kidney injury, *CHD* coronary heart disease, *COPD* chronic obstructive pulmonary disease, *DM* diabetes mellitus, *pts* points, *PAH* pulmonary arterial hypertension, *BMI* body mass index, *LVEF* left ventricular ejection fraction.

**Table 2:** Parameters of renal organ function in the overall cohort, in non-AKI and AKI patients.

	Total	Non-AKI (n = 20)	AKI (n = 30)	p value
Parameters of renal organ function (at admission and during in-hospital stay)				
Creatinine at admission (mg/dl)	1.39 ± 0.69	1.08 ± 0.46	1.6 ± 0.74	0.004
GFR at admission (ml/min)	59.02 ± 22.43	70.5 ± 26.01	43.27 ± 17.89	< 0.001
Lowest creatinine during in-hospital stay (mg/dl)	1.05 ± 0.46	0.87 ± 0.33	1.17 ± 0.51	0.01
Highest creatinine during in-hospital stay (mg/dl)	1.7 ± 0.76	1.21 ± 0.36	2.02.03 ± 0.78	< 0.001
Highest GFR during in-hospital stay (ml/min)	82.36 ± 31.5	96.15 ± 30.54	73.17 ± 29.11	0.005
Lowest GFR during in-hospital stay (ml/min)	47.82 ± 21.56	63.7 ± 20.51	37.23 ± 14.3	< 0.001
History of CKD n (%)	40 (80)	13 (65.0)	27 (90.0)	0.03
CKD stage G1 (n (%))	10 (20)	7 (33.3)	3 (10.3)	
	20 (40)	11 (52.4)	9 (31.0)	
	15 (30)	2 (9.5)	13 (44.8)	
	5 (10)	1 (4.8)	2 (13.8)	
AKI Stage 0 (n (%))	20 (40.0)	–	–	
AKI Stage 1	4 (8.0)	–	4 (13.3)	
AKI Stage 2	2 (4.0)	–	2 (6.7)	
AKI Stage 3	24 (48)	–	24 (80.0)	
RRT during in-hospital stay (n (%))	24 (48)	0 (0)	24 (82)	
Contrast agent (ml)	180 (80)	160 (70)	200 (98)	0.99

*AKI* acute kidney injury, *GFR* glomerular filtration rate, *CKD* chronic kidney disease, *AKI* acute kidney injury, *RRT* renal replacement therapy.

**Table 3:** Hemodynamic parameters of the overall Impella cohort, in Non-AKI and AKI patients.

	Total	Non-AKI (n = 20)	AKI (n = 30)	p value
Hemodynamic parameters (within the first 24 h after admission)				
SAP (mmHg)	110 ± 26	111 ± 32	109 ± 20	0.864
DAP (mmHg)	60 ± 11	60 ± 11	59 ± 11	0.892
MAP (mmHg)	86 ± 13	88 ± 13	85 ± 13	0.32
Heart rate (bpm)	100 ± 13	99 ± 14	101 ± 13	0.644
CO (l/min)	5.6 ± 2.02	5.95 ± 1.63	5.28 ± 2.24	0.258
CVP (mmHg)	9.9 ± 5.9	7.9 ± 5.1	12.33 ± 6.01	0.007
sPAP (mmHg)	35.1 ± 10.16	30.8 ± 7.0	37.9 ± 11.02	0.008
PCWP (mmHg)	13.3 ± 8.6	11.1 ± 6.5	14.9 ± 9.6	0.149
SVR (mmHg)	1031 ± 513	994 ± 331	1057 ± 609	0.675
PVR (mmHg)	231 ± 226	211 ± 198	244 ± 246	0.62
Lactate (mmol/l)	1.7 ± 1,6	1.3 ± 0.68	1.9 ± 1.96	0.07
SvO_2_ (%)	62.15 ± 14.9	66.9 ± 17.19	59.12 ± 12.63	0.07
Norepinephrine (µg/kg/min)	0.2 ± 0.33	0.14 ± 0.28	0.25 ± 0.36	0.313
Dobutamine (µg/kg/min)	4.14 ± 2.48	3.38 ± 2.0	4.71 ± 2.69	0.058

*AKI* acute kidney injury, *SAP* systolic arterial pressure, *DAP* diastolic arterial pressure, *MAP* mean arterial pressure, *CO* cardiac output, *CVP* central venous pressure, *sPAP* systolic pulmonary arterial pressure, *PCWP* pulmonary capillary wedge pressure, *SVR* systemic vascular resistance, *PVR* pulmonary vascular resistance, *SvO*_*2*_ central venous oxygen saturation.

### Prevalence of AKI and risk factors

40 (80%) patients had a known medical history of chronic kidney disease (CKD). In 10 (20%) patients CKD stadium G1 had been diagnosed and 20 (40%) patients had a known CKD stadium G2 whereas 15 (30%) and 5 (10%) individuals were accompanied by CKD stadium G3a and G3b respectively. At admission, the whole cohort had a mean creatinine of 1.39 ± 0.69 mg/dl and in 13 (26%) patients AKI was diagnosed according to 2012 KDIGO criteria whereas, after the first 48 h, a total of 30 (60%) patients developed AKI. 4 (8%) of these patients were classified at AKI stage1, 2 (4%) at AKI stage 2 and 24 (48%) at AKI stage 3. In 24 (48%) patients renal replacement therapy with continuous hemodiafiltration (CVVHDF) had to be conducted (Table 2).

Comparing the no AKI and AKI subgroups patients with AKI had a significantly higher SOFA and SAPS II score (6 (6) vs. 11 (5), p = 0.001 and 36.3 ± 11.9 vs. 50.17 ± 14.51, p = 0.001 respectively) (Table 1). Furthermore, AKI patients were accompanied by a significantly lower GFR at admission (43.27 ± 17.89 ml/min vs. 70.5 ± 26.01 ml/min, p < 0.001) as well as by a significantly reduced lowest GFR level (37.23 ± 14.3 ml/min vs. 63.7 ± 20.51 ml/min, p < 0.001) (Table 2).

On the other hand, no significant differences of SAP, MAP, heart rate, and CO were documented. Interestingly CVP was significantly elevated in the AKI subgroup (12.33 ± 6.01 vs. 7.9 ± 5.1, p = 0.007) while the tissue perfusion-related values of lactate and SvO_2_ were reduced in AKI individuals (1.9 ± 1.96 mmol/l vs. 1.3 ± 0.68 mmol/l, p = 0.07 and 59.12 ± 12.63% vs. 66.9 ± 17.19%, p = 0.07 respectively) but p values missed slightly statistical significance. Furthermore, regarding dosages of vasopressors and inotropics, no relevant differences between the two subgroups were documented (Table 3).

In the univariate analysis, a highly significant association of chronic kidney disease (CKD) history (OR 4.846, p = 0.04), CVP at admission (OR: 1.185, p = 0–014), SvO_2_ at admission (OR 0.942, p < 0.001) and GFR at admission GFR at admission (OR 0.951, p = 0.002) with the development of AKI could be demonstrated. Moreover, multivariate analysis revealed CVP at admission (OR 1.216, p = 0.020), and SvO_2_ at admission (OR 0.93, p = 0.029) as independent predictors for the onset of AKI. Due to the collinearity of the onset of AKI and GFR at admission, GFR was not included in the multivariate analysis (Table 4).

**Table 4:** Risk factors of acute kidney injury (AKI).

	Univariate analysis			Multivariate Analysis		
	OR	95% CI	P value	OR	95% CI	p value
Gender (male)	0.523	0.446–6.583	0.432			
Age (years)	1.032	0.982–1.08	0.247			
History of CKD	4.846	1.075–21.84	0.04			
History of DM	1.657	0.495–5.54	0.412			
History of aHT	2.25	0.445–11.37	0.326			
LVEF (%)	0.966	0.917–1.017	0.183			
HR (bpm)	1.01	0.968–1.06	0.637			
MAP (mmHg)	0.977	0.932–1.356	0.316			
CVP (mmHg)	1.185	1.035–1.166	0.014	1.216	1.031–1.434	0.020
CO (l/min)	0.844	0.627–1.137	0.844			
SVR	1.0	0.999–1.001	0.669			
PVR	1.0	0.998–1.003	0.615			
PAPi	0.883	0.742–1.051	0.161			
PCWP	1.063	0.977–1.157	0.156			
meanPAP	1.018	0.972–1.066	0.456			
Lactate (mmol/l)	1.871	0.798–4.391	0.15			
SvO_2_ (%)	0.955	0.907–1.006	0.081	0.930	0.872–0.993	0.029
GFR at baseline	0.951	0.920–0.983	0.003	0.928	0.885–0.973	0.002

Simple and multiple regression analysis of patient characteristics, disease history and admission parameters (95% CI: 95% Confidence Interval, OR: Odds Ratio).

*aHT* arterial hypertension, *CKD* chronic kidney disease, *DM* diabetes mellitus, *HR* heart rate, *LVEF* left ventricular ejection fraction, *MAP* mean arterial pressure, *CVP* central venous pressure, *CO* cardiac output, *SvO*_*2*_ central venous oxygen saturation, *GFR* glomerular filtration rate.

### Analysis of survival

**Figure 1:**
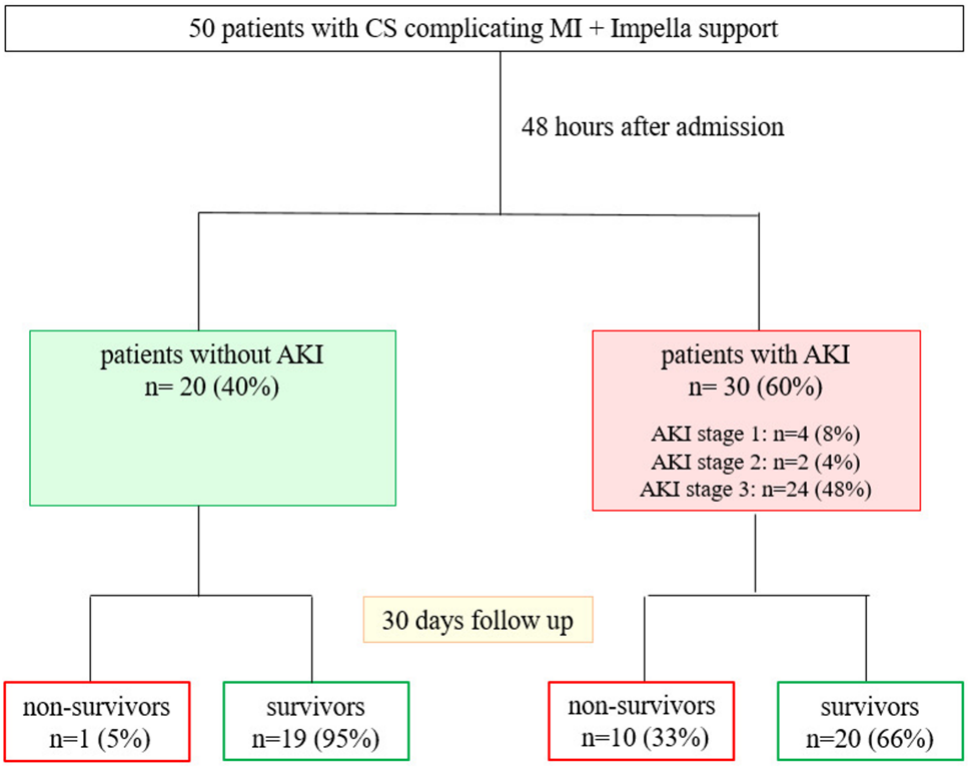
Study cohort.

**Figure 2:**
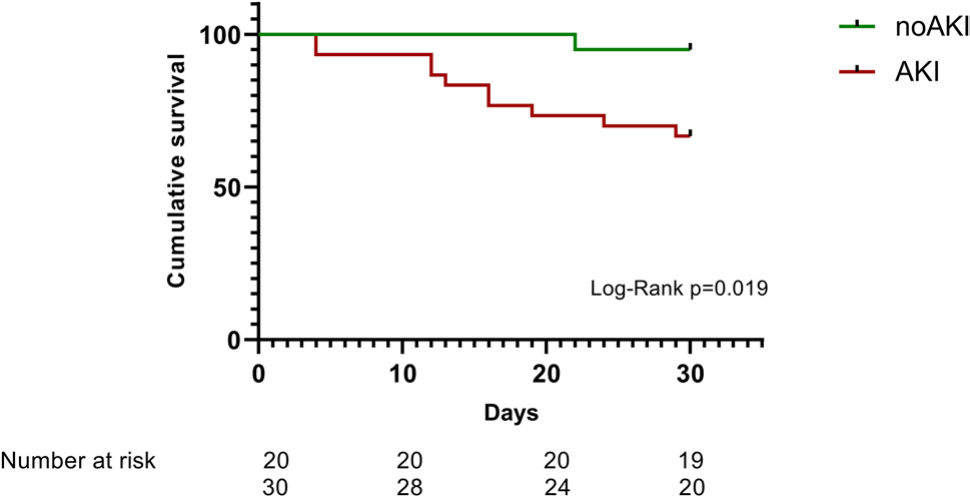
30-Day cumulative survival. Red: patients with acute kidney injury (AKI), green: patients without AKI (Non-AKI) (Kaplan–Meyer analysis).

**Figure 3:**
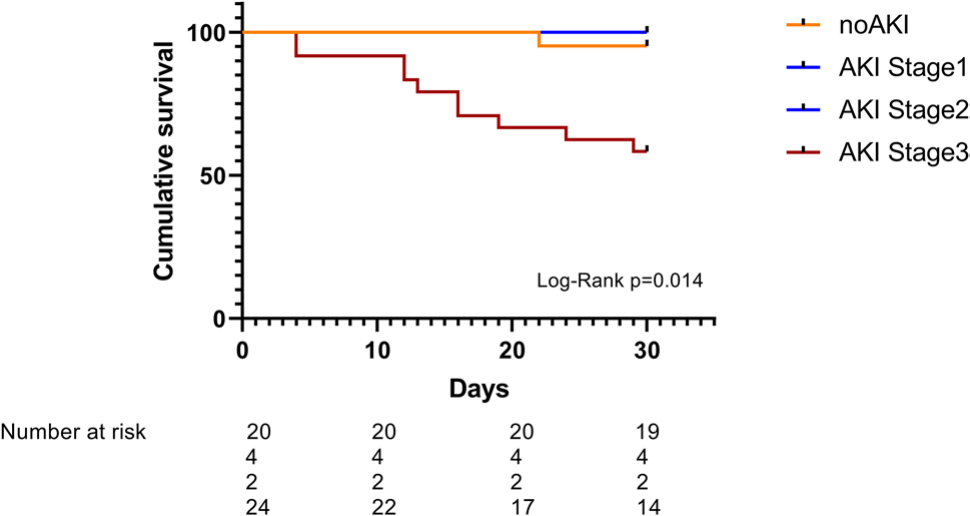
30-Day cumulative survival. Orange: no kidney injury, blue: AKI stages 1 and 2, purple: AKI stage 3 (Kaplan–Meyer analysis).

The 30-day mortality of the whole cohort was 22%. Patients with AKI were accompanied by a significantly higher 30-day mortality than patients without AKI (n = 10/30 (33%) vs. n = 1/20 (5%); p = 0.019). The overwhelming percentage of diseased patients was in AKI stadium 3 (n = 10/24; 41, 6%) and, consequently, this subgroup was accompanied by an even higher 30-day mortality rate (Figs. 1, 2 and 3).

In the univariate analysis, lactate at admission (HR 1.489, p = 0.004), the dosage of norepinephrine (HR 1.027, p = 0.012), and AKI stage 3 (HR 0.074, p = 0.013) were significantly associated with survival. GFR at admission was also strongly associated with 30-day mortality but the p-value did not reach statistical significance (p = 0.054). In the Cox regression analysis, AKI stage 3 (HR = 0.095, p = 0.026) and norepinephrine dosage (HR 1.027, p = 0.008) were independently associated with 30-day mortality (Table 5).

**Table 5:** Predictors of 30-day mortality.

	Univariate analysis			Multivariate analysis		
	HR	95% CI	p value	HR	95% CI	p value
Gender (male)	2.41	0.646–8.991	0.19			
Age (years)	1.027	0.969–1.089	0.362			
History of CKD	0.846	0.176–4.077	0.846			
History of DM	0.76	0.19–3.038	0.697			
History of aHT	0.555	0.115–2.673	0.463			
AKI Stadium 3	9.5	1.169–77.36	0.035	0.095	0.012–0.753	0.026
LVEF (%)	1.204	0.967–1.804	0.423			
HR (bpm)	0.987	0.939–1.038	0.617			
MAP (mmHg)	1.004	0.954–1.058	0.871			
CVP (mmHg)	0.957	0.845–1.085	0.492			
CO (l/min)	1.006	0.688–1.470	0.976			
Lactate (mmol/l)	1.489	1.139–1.947	0.004			
SvO_2_ (%)	0.986	0.951–1.022	0.449			
GFR	0.955	0.926–0.984	0.003			
Norepinephrine dosage (µg/kg/min)	1.027	1.006–1.048	0.012	1.027	1.007–1.048	0.008

Cox regression analysis (univariate and multivariate) of patient characteristics, disease history, admission parameters and treatment (*95% CI* 95% confidence interval, *OR* odds ratio).

*aHT* arterial hypertension, *CKD* chronic kidney disease, *DM* diabetes mellitus, *LVEF* left ventricular ejection fraction, *MAP* mean arterial pressure, *CVP* central venous pressure, *CO* cardiac output, *SvO*_*2*_ central venous oxygen saturation, *GFR* glomerular filtration rate.

## Discussion

We here defined early predictors for the occurrence of AKI during Impella support, which may be useful to optimize therapy control in CS. To our knowledge, this is the first study to analyze the occurrence of AKI according to the KDIGO criteria in CS patients during Impella support, including the evaluation of a wide range of patient characteristics, invasively obtained hemodynamics, laboratory parameters, and other indices that may influence renal function in shock.

In our Impella cohort, the prevalence of AKI was 60%, in accordance with other published data ^3,17,18^. The development of AKI was significantly more common in patients with CKD, possibly reflecting a reduced compensatory ability caused by the development of chronic renal organ damage. Moreover, as expected, patients developing AKI had a significantly lower GFR at admission.

Ultimately, three independent and early predictors of AKI could be defined in our cohort, namely GFR, CVP, and SvO_2_. While pre-existing patient-associated parameters such as CKD and thus reduced GFR on admission cannot be directly influenced, the two other hemodynamic parameters CVP and SvO2 can be improved by therapeutic methods (fluid management, catecholamines) and possibly also by MCS such as the Impella.

According to our data, hemodynamic parameters that reflect myocardial function and organ and tissue perfusion in CS seem to play an outstanding role in the occurrence of AKI and renal organ failure. However, the quality and effectiveness of the interaction between the individual treatment options used and the parameters monitored and adjusted, could ultimately determine the outcome and be decisive for the preservation of organ function in CS.

In patients with AKI, in univariate analysis, SvO_2_ was noteworthy lower and, correspondingly, although the p-value moderately missed statistical significance, lactate level was found to be considerably higher compared to non-AKI patients indicating an impaired tissue and end-organ perfusion as a consequence of low cardiac output. While AKI patients presented no significant differences of MAP and PCWP, CVP and sPAP on the other hand were notably elevated reflecting a substantial venous congestion and right ventricular burden. Whereas the elevated CVP and sPAP in AKI patients could pathophysiological be attributed to systemic overload in the situation of compromised hemodynamics as well as to oliguria or anuria in CS, the left ventricular unloading with the Impella seems to effectively support left ventricle function since PCWP values of patients with and without AKI presented comparable.

In addition, the vicious circle of impaired tissue and organ perfusion, which may also be caused by increased venous congestion, leads to the activation of a cascade of neuro-hormonal and inflammatory mechanisms that further deteriorate organ function ^19–21^. Moreover, in combination with oxidative stress, the so-called glycosaminoglycan (CAG) networks could be damaged ^22^. The CAGs, which are responsible for buffering sodium, influence endothelial function. An impaired function of the CAGs leads to an elevation of vascular resistance and imbalanced endothelial nitric oxide production. The resulting endothelial dysfunction requires an increased workload for both, the left and right myocardial ventricle, further impairing end-organ perfusion ^23^. Thus, in case, right ventricular filling pressure increases for example in a situation of CS complicating right ventricular MI, a backward failure may lead to a further increase of CVP. Furthermore, also left ventricular failure with consecutive congestion could cause pulmonary edema with secondary deterioration of right ventricular function and consecutive abdominal organ congestion. Among all complications of AKI, volume overload aggravating venous congestion seems to be the one with the greatest impact on mortality ^24^. At the same time, recent data emphasize that venous congestion and CVP are also one of the most important pathomechanisms for the development of CKD ^25–32^. This seems in line with to pathophysiological understanding, as a backward failure can usually also be accompanied by a forward failure with consecutive organ minder perfusion. According to the findings of this study cohort, the elevated CVP could be attributed to left ventricular systolic function impairment with consecutive post capillary congestion leading to elevated sPAP burdening right ventricular function ultimately increasing venous congestion.

Therefore, in our opinion, the reduction of venous congestion through diuretics or early RRT while improving left ventricular function and organ perfusion, possibly by additional Impella support may be beneficial for a more favourable outcome.

As mentioned above, SvO_2_ at admission could be identified as an independent and early predictor for the occurrence of AKI. This association underscores the relevance of adequate organ and tissue perfusion in the situation of CS to avoid end-organ failure. The use of catecholamines and vasopressors in CS often temporarily stabilizes blood pressure at the expense of severely increasing systemic peripheral vascular resistance and cardiac afterload ^33–35^. Interestingly, as we were able to show, AKI stage 3 and norepinephrine dosages were independent predictors of 30-day mortality. Therefore, the reduction of vasopressor doses during CS should be aimed for. In this regard, the use of MCS such as the Impella is an option that may be considered in hemodynamically unstable patients, as during Impella support the CO and SvO_2_ increase ^9^.

As it is known from the current literature, in the situation of CS, the 30-day mortality remains higher than 50% without any noteworthy changes in the last two decades ^3^. The 30-day mortality of our cohort, however, was lower at 22%. This may be attributed to the fact that the patients of this cohort received early hemodynamic support with left ventricular Impella implemented according to standard algorithms in CS. However, the high SOFA score of this cohort of 10 (IQR:5) with an expected mortality between 33 and 50%, which is in line with the known mortality rates in the current literature contradicts this explanation at first glance ^36^. On the other side, one may postulate, that the interaction of myocardial function and hemodynamic support by MCS may improve the negative prognosis predicted by the scores.

In summary, early targeted monitoring of relevant parameters, as demonstrated here, should be recommended to enable timely adaptation of therapeutic approaches, in particular relieving venous congestion, increasing CO, and reducing peripheral vascular resistance to improve tissue and organ oxygenation, which in turn may reduce the prevalence of AKI ^37–39^. The use of MCS may also be supportive in this respect ^40^.

## Limitations

The relatively small number of patients and the single center experience belong to the limitations of this study. Furthermore, the retrospective analysis also limits the interpretation of the data. However, to our knowledge, this is the first study to analyze early and subsequent predictors of AKI according to the KDIGO criteria in CS patients with Impella support using a broad spectrum of parameters and characteristics. To further evaluate the clinical relevance of these data, larger prospective randomized studies are needed that include a broader cohort of patients with different stages of CS as defined by the SCAI classification.

## Conclusions

Renal function is one of the most relevant key factors regarding the prognosis of patients with CS and AKI deteriorates outcome dramatically. Increased venous congestion, reduced SvO_2,_ and GFR on admission are early independent predictors for AKI complicating CS in patients supported with Impella, while catecholamine dosages and the onset of AKI are independent predictors of mortality. The reduction of catecholamine dosages during ongoing support with pVLAD like the Impella and the decrease of venous congestion by additional volume restriction and if indicated the early use of RRT may improve prognosis.

### Supplementary Information

Supplementary Information.

## Data Availability

Data will be made available upon individual request (contact: corresponding author).
