# Supplementary material for: Early risk predictors of acute kidney injury and short-term survival during Impella support in cardiogenic shock
Source: Sci Rep. 2024 Jul 30;14:17484. doi: 10.1038/s41598-024-68376-w (PMC11289486; doi:10.1038/s41598-024-68376-w)
Supplement: Supplementary file 1 — Supplementary Information. [file 41598_2024_68376_MOESM1_ESM.pdf]

Suppl. Table 1. SCAI classification and data about PCI strategy of the overall cohort

SCAI classification	A: n=0 (0%)<br>B: n=0 (0%)<br>C: n=50 (100%)<br>D: n=0 (0%)<br>E: n=0(0%)
Cardiac arrest	n=0 (0%)
Culprit lesion intervention	n=50 (100%)
Vessels intervened	LMCA: n= 4 (8%)<br>LAD: n=19 (38%)<br>LAD/RD1: n=6 (12%)<br>LAD/RD2: n=4 (8%)<br>LCX: n=8 (16%)<br>RIM: n=6 (12%)<br>RPLD: n=3 (6%)

Abbreviations: SCAI: Society for Cardiovascular Angiography and Interventions, LMCA: left main coronary artery, LAD: left anterior descending, RD1: diagonal branch 1, RD2: diagonal branch 2, LCX: left circumflex artery, RIM: intermediary branch, RPLD: right posterolateral branch.
